# Analysing Antibodies Against Respiratory Viruses in Breast Milk: A Pilot Study

**DOI:** 10.3390/v18060593

**Published:** 2026-05-24

**Authors:** Sindre H. Hauan, Camilla H. Nundal, Sarah Lartey Jalloh, June Skudal, Elin Ekornes Håskjold, Sigrid Christiansen Bøe, Camilla Tøndel, Linn Marie Sørbye, Rebecca J. Cox, Karl A. Brokstad

**Affiliations:** 1 Department of Safety, Chemistry and Biomedical Laboratory Sciences, Western Norway University of Applied Science, Postboks 7030, 5020 Bergen, Norway; sindre.haukefer.hauan@hvl.no (S.H.H.); camilla.hatlevoll.nundal@hvl.no (C.H.N.); 672455@stud.hvl.no (J.S.); elinekhaa@hotmail.com (E.E.H.); 573062@stud.hvl.no (S.C.B.); 2 Influenza Centre, Department of Clinical Science, University of Bergen, 5020 Bergen, Norway; sarah.lartey@uib.no (S.L.J.); rebecca.cox@uib.no (R.J.C.); 3 Department of Paediatrics, Haukeland University Hospital, 5020 Bergen, Norway; camilla.tondel@uib.no; 4 Department of Clinical Science, University of Bergen, 5020 Bergen, Norway; 5 Department of Obstetrics and Gynaecology, Haukeland University Hospital, 5020 Bergen, Norway; linn.marie.sorbye@hvl.no; 6 Faculty of Health and Social Sciences, Western Norway University of Applied Sciences, 5020 Bergen, Norway; 7 Department of Microbiology, Haukeland University Hospital, 5020 Bergen, Norway

**Keywords:** respiratory viruses, influenza virus, coronavirus, RSV, breast milk, immunoglobulins, antibodies, immunity

## Abstract

Background: Lower respiratory tract infections remain a major cause of morbidity and mortality in infants worldwide. Newborns possess an immature immune system but acquire passive immunity through maternal antibodies transferred via the placenta (IgG) and breast milk (IgA). Maternal vaccination may enhance this protection. This study aimed to quantify antibody levels against respiratory viruses in serum and breast milk from lactating women. Methods: Serum and breast milk samples were collected from 26 lactating mothers. Antibody levels were measured using an indirect enzyme-linked immunosorbent assay (ELISA) targeting seven viral antigens: influenza A (A/Thailand, A/California), influenza B (B/Phuket, B/Austria), SARS-CoV-2 (Spike and receptor-binding domain, RBD) and RSV F pre-fusion protein. Antibody isotypes IgG, IgA and IgM were analysed. Results: Virus-specific IgG and IgA antibodies were detected in all samples. Breast milk showed the highest levels of IgA, whereas serum contained higher IgG levels. A moderate positive correlation was observed between serum and milk IgG. No correlation was found between serum IgG and milk IgA, but both levels were elevated. Conclusions: Breast milk and serum contain relatively high levels of antibodies against the tested respiratory viruses. The elevated levels of serum IgG and milk IgA indicate a coordinated defence between systemic and mucosal immunity in response to infections. The levels and correlation of specific isotypes point to the source of the antibodies: milk IgG probably originates from the blood, whereas milk IgA is produced locally.

## 1. Introduction

According to the World Health Organisation (WHO), respiratory tract infections remain a major global health challenge and are among the leading causes of morbidity and mortality, particularly in vulnerable populations such as infants and young children [1]. Lower respiratory tract infections are consistently ranked among the top causes of death worldwide according to global health estimates and represent a significant burden in both low- and middle-income countries as well as high-income settings [2,3]. Although mortality rates have declined over recent decades, respiratory infections continue to result in substantial hospitalization rates and long-term health consequences, particularly in children under five years of age [2,3].

Breast milk is a complex, nutrient-rich fluid that provides all the essential components for infant growth and optimal health and confers protection against common infectious agents [4,5,6,7]. Infants are particularly susceptible to respiratory infections due to their naive immune systems, which leads to a reduced capacity to mount effective immune responses against pathogens. As a result, newborns rely heavily on passive immunity provided by maternal antibodies [8,9,10,11,12]. This passive protection is mediated through two primary routes: transplacental transfer of immunoglobulin G (IgG) during pregnancy and postnatal transfer of immunoglobulin A (IgA) through breast milk. Together, these mechanisms provide critical early-life protection against infectious agents during a period when the infant’s own immune system is still developing. Among the different isotypes, IgG is the most abundant antibody in circulation and is essential for systemic immunity, including the neutralization of pathogens and activation of immune effector mechanisms. Importantly, IgG can cross the placenta during the third trimester, providing the foetus with a repertoire of maternal antibodies that can persist for several months after birth [13,14,15]. In contrast, IgA, particularly secretory IgA (sIgA), is the dominant immunoglobulin at mucosal surfaces and is highly abundant in breast milk. sIgA functions primarily by preventing pathogen adherence to epithelial cells and neutralizing viruses at mucosal entry sites without inducing inflammation, making it especially important in protecting the respiratory and gastrointestinal tracts of infants [16,17].

The composition of breast milk is dynamic and reflects the immunological experiences of the mother, including prior infections and vaccinations. Antigen-specific B cells activated in mucosal tissues can migrate to the mammary gland, where they differentiate into plasma cells that produce IgA [18,19]. This process results in the secretion of pathogen-specific sIgA into breast milk, thereby transferring targeted immune protection to the infant. Consequently, breast milk serves not only as a source of nutrition but also as an important immunological interface between mother and child.

Maternal vaccination has emerged as a key strategy to enhance passive immunity in infants. Vaccination during pregnancy has been shown to increase maternal IgG levels, which are subsequently transferred across the placenta to the foetus [20,21]. More recently, attention has also been directed toward the role of vaccination in influencing antibody levels in breast milk. Several studies have demonstrated that vaccination against pathogens such as SARS-CoV-2 can increase levels of specific antibodies in breast milk [22,23], suggesting an additional pathway for protecting infants during breastfeeding.

Respiratory viruses such as influenza A and B viruses, respiratory syncytial virus (RSV) and severe acute respiratory syndrome coronavirus 2 (SARS-CoV-2) represent significant causes of respiratory illness across all age groups. Influenza viruses are characterised by high mutation rates and seasonal variability, necessitating regular vaccine updates. SARS-CoV-2, the causative agent of the COVID-19 pandemic, has highlighted the importance of understanding immune protection mechanisms, particularly in populations that cannot be directly vaccinated, such as very young infants. While vaccination strategies have been widely implemented, the extent to which maternal antibodies, particularly those present in breast milk, contribute to infant protection against these viruses remains incompletely understood.

Despite growing evidence supporting the presence of pathogen-specific antibodies in breast milk, quantitative data comparing antibody levels across respiratory viruses and immunoglobulin isotypes within the same cohort remain limited. Furthermore, the relationship between systemic antibody levels in maternal serum and mucosal antibody levels in breast milk remains poorly elucidated. Understanding this relationship is essential for evaluating the potential impact of maternal vaccination and infection history on infant immunity. By providing both methodological advances and comparative immunological data, this pilot study aims to advance understanding of passive immunity in early life and inform future strategies for maternal vaccination and infant protection against respiratory infections.

## 2. Materials and Methods

### 2.1. Study Design and Participants

This cross-sectional pilot study investigated antibody levels against respiratory viruses in paired serum and breast milk samples from lactating women.

A total of 26 healthy lactating mothers aged ≥18 years were recruited from Western Norway between September and November 2025. Inclusion criteria required participants to be actively breastfeeding and to have an infant aged less than 12 months. Participants were recruited through social media platforms, including Ammehjelpen (Norwegian breastfeeding support network; “https://ammehjelpen.no/ (accessed on 21 April 2026)”), as well as through voluntary self-enrolment via snowball sampling and a project-specific website (Morsmelkprosjektet; “https://www.morsmelk.net (accessed on 21 April 2026)”).

Participants provided further background information through a questionnaire, including maternal age, infant age, history of respiratory infections in the past three years, and vaccination status (influenza and COVID-19). The cohort included both vaccinated and unvaccinated individuals, allowing for exploratory comparisons of immune responses.

### 2.2. Sample Collection and Storage

Each participant provided paired biological samples: 5 mL of peripheral venous blood and 5 mL of breast milk. The samples were immediately processed. Blood samples were collected in serum separator tubes and allowed to clot prior to centrifugation, and serum was extracted. Breast milk samples were self-expressed by participants on the day of sampling. Both serum and breast milk samples were aliquoted into fractions of 500 µL each to minimize freeze–thaw cycles. All samples were biobanked at −80 °C in an ultrafreezer at the Western Norway University of Applied Sciences until analysed in bulk.

### 2.3. Sample Processing

Prior to analysis, samples were thawed at 4 °C. Serum samples were analysed within 7 days of thawing, while breast milk samples were analysed within 15 days. To reduce interference from lipids and cellular components, breast milk samples were centrifuged at 800× *g* for 15 min at room temperature. The aqueous phase beneath the lipid layer was carefully collected and transferred to new tubes for analysis. Although preliminary testing indicated minimal differences between centrifuged and non-centrifuged samples, centrifugation was applied consistently to standardise sample preparation.

### 2.4. Antigens and Target Proteins

Antibody responses were analysed against seven viral antigens representing common respiratory pathogens—Influenza viruses: A/California/07/2009 (H1N1), A/Thailand/08/2022 (H3N2), B/Phuket/3073/2013 (Yamagata lineage) and B/Austria/1359417/2021 (B/Victoria lineage); SARS-CoV-2: full-length Spike protein and receptor-binding domain (RBD); RSV: (A2) the F pre-fusion protein. The influenza and coronavirus antigens were purified in house, while the RSV antigen was purchased (RSV-F0-30p; eEnzyme^®^, Rockville, MD, USA).

### 2.5. Enzyme-Linked Immunosorbent Assay (ELISA) Procedure

An indirect ELISA was used to detect antigen-specific antibodies. High-binding 96-well polystyrene microtiter plates (Nunc MaxiSorp, Roskilde Denmark) were coated with antigen solutions and incubated overnight at 4 °C to allow for adsorption. Following incubation, plates were washed with phosphate-buffered saline containing 0.05% Tween-20 (PBS-T) to remove unbound antigens. Non-specific binding sites were blocked using a PBS blocking solution. For serum sample analyses with influenza antigens, we used 5% dry milk powder and 1% bovine serum albumin (BSA); for SARS-CoV-2 antigens, we used 3% milk in the blocking buffer and 1% milk in the sample diluent. For breast milk sample analyses, we used 3% BSA (no milk) for all antigens, including RSV (for both serum and breast milk).

Samples were serially diluted in a five-fold dilution series. Starting dilutions for serum IgG and IgA were 1:100 for SARS-CoV-2 and 1:50 for influenza and RSV. Serum IgM dilutions started at 1:50 and breast milk IgG, IgA and IgM at 1:50. Diluted samples were added to antigen-coated wells and incubated to allow for antigen–antibody binding. Following washing, horseradish peroxidase (HRP)-conjugated secondary antibodies specific for human immunoglobulins were added. After incubation and washing steps, tetramethylbenzidine (TMB) substrate was added. Enzymatic conversion of TMB by HRP produced a colorimetric signal proportional to antibody concentration. The reaction was stopped using 0.5 M hydrochloric acid (HCl) and absorbance was measured at 450 nm (signal) and 620 nm (background correction) using a Synergy H1 hybrid microplate reader (BioTek, Agilent Technologies, Santa Clara, CA, USA).

### 2.6. Endpoint Titre Determination and Statistical Analysis

To allow for quantitative comparison between samples, endpoint titres were calculated from serum and breast milk dilution curves. Background-corrected absorbance values were used to determine the highest dilution at which the signal exceeded a predefined cut-off value (absorbance = 0.2). Endpoint titre was defined as the highest dilution yielding a positive signal above the cut-off threshold. This method reduces the impact of assay variability and matrix effects, providing a robust measure of antibody concentration. Raw absorbance data were exported from Gen5 software (version 2.00.18) and processed using Microsoft Excel. Background correction was performed by subtracting the absorbance at 620 nm from that at 450 nm. Statistical analyses and graphs were prepared using R (version 4.5.2), RStudio (version 2026.01.0-392), and the packages tidyverse (v.2.0.0), readxl (v.1.4.5), minpack.lm (v.1.2-4) and corrplot (v.0.95). Pearson correlation analysis was performed using the cor() function in the built-in {stats} package. We defined the correlation coefficient |R| as no (0.00–0.09), low (0.10–0.39), moderate (0.40–0.69) and high (0.70–1.00). All reported correlation coefficients given had a *p*-value less than 0.05. R-scripts are available on request.

## 3. Results

### 3.1. Samples and Vaccine Status

Blood and breast milk were collected from 26 lactating women (range 26–40, median 32 years) at our sampling clinic at the University of Western Norway. Vaccination status for the last three years prior to sampling was recorded. Eighteen of the subjects had a recent influenza vaccine, three had vaccines against SARS-CoV-2 and influenza in the last three years, and five were unvaccinated during the last 3 years. The high vaccine coverage was mainly due to the majority of mothers working in the health system. Health care workers are encouraged to be vaccinated and receive free vaccines.

### 3.2. Endpoint Titres

**Figure 1:**
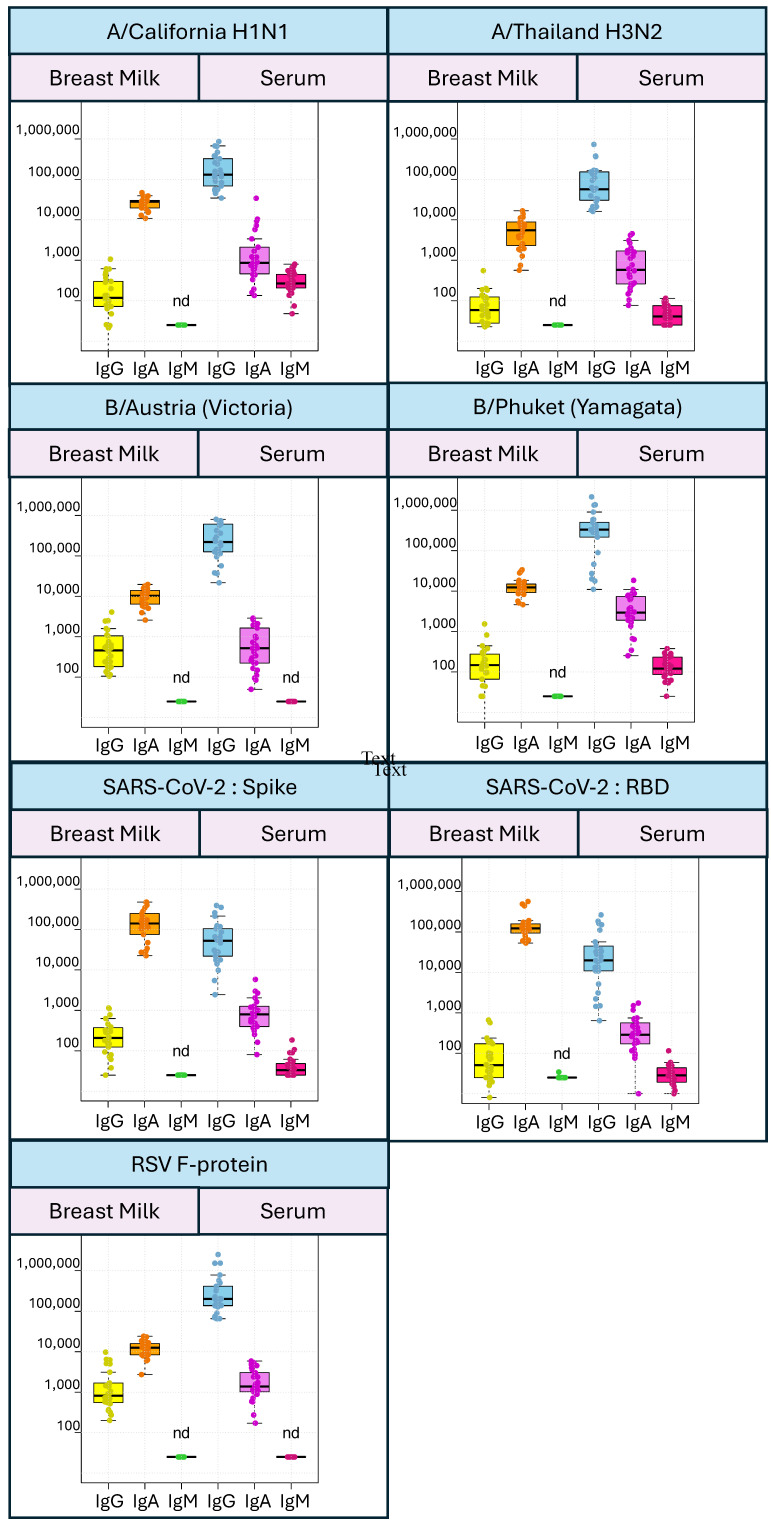
Endpoint titre boxplot. Serum and breast milk samples were tested against influenza, SARS-CoV-2, and RSV antigens by ELISA in serial dilutions. The endpoint titres against the antigens were calculated and presented in this hybrid boxplot–strip chart. The median and quartiles are indicated in the boxplot, and the individual samples are indicated as coloured dots. The specific antibody isotypes IgG, IgA and IgM were measured individually in serum and breast milk. The virus antigen and sample type are indicated at the top of each panel. To the left is endpoint titres on a log10 scale, and at the bottom, the antibody isotype is indicated. nd = not detected.

We observed that many samples gave high readings on the microplate reader at high concentrations (i.e., low dilution ratios) (Figure 1). This was particularly pronounced for serum IgG and breast milk sIgA. We were able to measure IgG, IgA, and IgM in most serum and breast milk samples, but the IgM signals were generally so low that endpoint titres could not be calculated; therefore, these data were not processed further. Endpoint titres are a good method for calculating semi-quantitative signals because they take into account all measurement points in the dilution series and statistically determine dilution/concentration at a given threshold, rather than relying on a single-point measurement.

### 3.3. Influenza Virus Antibodies

At first glance (Figure 1), the results from the endpoint measurement appear similar (same pattern), but subtle differences can be linked to vaccination and infections. In analyses against influenza, we observed that serum IgG was high (endpoint titre: 120,106–464,468), and although breast milk IgA was elevated (ept: 6169–26,559), it was not at the same level. The high serum IgG levels may result from repeated influenza vaccinations that this cohort has received. Every year, about 10% of the population gets influenza, i.e., on average, a person will experience an influenza infection about every 10 years. This may possibly explain the lower IgA levels. An infection usually stimulates mucosal immunity, in which IgA is dominant.

### 3.4. Coronavirus (SARS-CoV-2) Antibodies

The SARS-CoV-2 analyses gave a slightly different pattern (Figure 1). Most people have received several (three to four) doses of vaccines since the pandemic, as we see in the high IgG levels in serum (ept: 86,410 and 48,614). Although the vaccine does not confer sterile immunity, it significantly reduces morbidity. Many have had COVID-19 infection in the aftermath of the pandemic, either sub-clinically or with a very mild course of the disease. This may explain the higher IgA levels in breast milk (ept: 179,874 and 160,473).

### 3.5. Respiratory Syncytial Virus (RSV) Antibodies

The RSV results are interesting, with relatively high serum IgG (ept: 422,503) and low milk IgA levels (ept: 12,543) (Figure 1). RSVs are commonly circulating and often cause mild or sub-clinical symptoms in adults. To our knowledge, none of the participants in the cohort were vaccinated against RSV.

### 3.6. Sample Correlation

**Figure 2:**
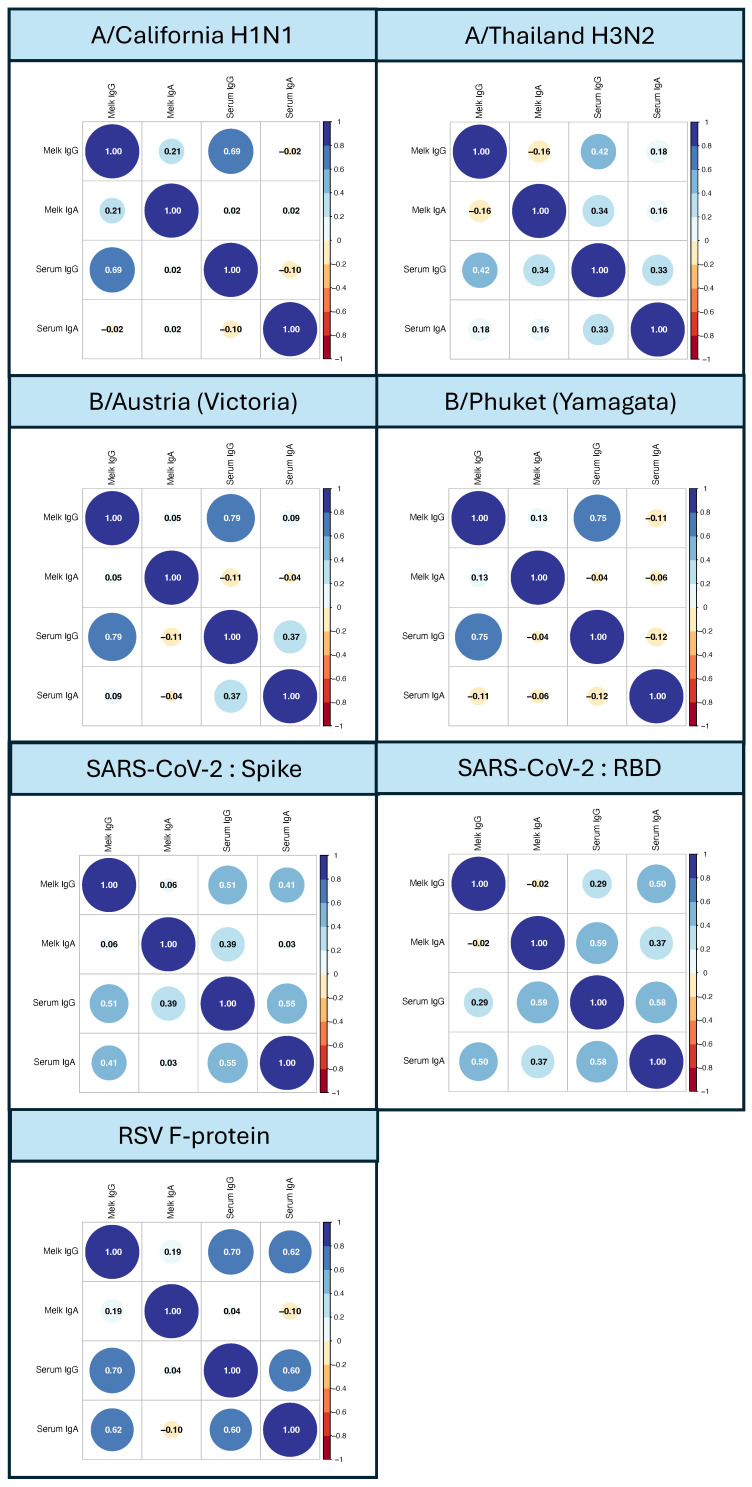
Correlation plots. Cross-correlation plots, comparing the endpoints for serum and breast milk isotypes IgG and IgA. IgM was not included in the analysis due to low or undetectable levels. The viral antigens are indicated above each panel, and IgG and IgM from serum and breast milk are indicated over and to the left of each correlation plot. The correlation (R) coefficient is indicated by numbers, and by the size and colour of the circles.

To examine the relationship between antibody isotypes in breast milk and serum, we performed a cross-correlation analysis (Figure 2). We omitted the IgM isotype from the analysis because levels were very low or absent and are probably of insignificant biological function. The analysis showed a moderate correlation between serum IgG and breast milk IgG against influenza viruses (correlation (R): 0.42–0.79, *p* < 0.05). SARS-CoV-2 also showed a moderate correlation between serum IgG (R: 0.51, 0.41, *p* < 0.05) and IgA (R: 0.29, 0.50, *p* < 0.05) and breast milk IgG. For the RSV virus, there was a moderate correlation between milk IgG and serum IgA (R: 0.62, *p* < 0.05) and IgG (R: 0.70, *p* < 0.05). Overall, there was a moderate correlation between breast milk IgG and serum IgG, and, in some cases, serum IgA. There was no correlation between serum IgG and breast milk IgA.

## 4. Discussion

This pilot study demonstrates that breast milk from lactating mothers contains specific antibodies against major respiratory viruses, including influenza A, influenza B, SARS-CoV-2 and RSV. The findings confirm that breast milk constitutes an important source of passive mucosal immunity for infants and further suggest a relationship between systemic and mucosal immune responses.

The predominance of IgA in breast milk observed in this study is consistent with the established role of secretory IgA (sIgA) as the dominant immunoglobulin at mucosal surfaces [16]. sIgA plays a crucial role in immune exclusion by neutralising pathogens and preventing their attachment to epithelial cells without inducing inflammation. This is particularly important in infants, where excessive inflammatory responses may be harmful due to the immaturity of the immune system.

In contrast, IgG was found at higher levels in serum, reflecting its role in systemic immunity. The differential distribution of immunoglobulin isotypes between serum and breast milk aligns with known immunological compartmentalisation [13,14]. However, the presence of IgG in breast milk, although at lower levels than IgA, may still contribute to pathogen neutralisation and immune protection.

The detection of virus-specific antibodies in all samples suggests prior exposure to the virus through infection and/or vaccination among participants. This reinforces the concept that maternal immunological history is directly reflected in the antibody composition of breast milk, thereby shaping infant immunity.

One of the key findings of this study is the elevated levels of serum IgG and breast milk IgA against the selected viruses, influenza A and B, SARS-CoV-2 and RSV. This relationship is not statistically correlated, but it supports the concept of a coordinated immune response between systemic and mucosal compartments.

A plausible biological explanation lies in the migration of antigen-activated B cells [18,19]. Following antigen exposure, either through infection or vaccination, B cells activated in mucosal-associated lymphoid tissues (MALTs) or in the systemic circulation may migrate to the mammary gland during lactation. There, they differentiate into plasma cells that produce dimeric IgA, which is subsequently transported into breast milk via the polymeric immunoglobulin receptor (pIgR).

This mechanism suggests that elevated systemic IgG levels may serve as an indirect marker of an enhanced mucosal IgA response, although the relationship is unlikely to be strictly linear. Factors such as antigen type, route of exposure, timing, and individual immune variability may influence this association.

The findings have important implications for maternal vaccination strategies. If increased serum IgG levels induced by vaccination are associated with increased IgA levels in breast milk, vaccination during pregnancy or lactation may provide dual benefits, direct protection of the mother and indirect protection of the infant via passive immunity. This is particularly relevant for respiratory viruses such as influenza and SARS-CoV-2, where infants are at increased risk of severe disease but may not be eligible for vaccination in early life [13]. Previous studies have demonstrated that maternal vaccination can significantly reduce hospitalization rates in infants [19]. The present findings support these observations by suggesting a mechanistic basis for enhanced antibody transfer through breast milk. However, the timing of vaccination may be critical. Vaccination during pregnancy primarily enhances transplacental IgG transfer, whereas vaccination during lactation may preferentially enhance mucosal IgA responses. The optimal timing and combination of these strategies warrant further investigation.

The findings are in agreement with previous studies demonstrating the presence of virus-specific antibodies in breast milk following infection or vaccination [24]. In particular, the strong IgA response aligns with studies highlighting the importance of mucosal immunity in early life protection. For SARS-CoV-2, recent research has shown that maternal vaccination leads to detectable antibody levels in breast milk and may reduce the risk of infant hospitalisation [19]. The present study extends these findings by demonstrating a correlation between serum and breast milk antibody responses. However, few studies have directly quantified and compared multiple respiratory virus antigens across both serum and breast milk within the same cohort. This study therefore contributes novel comparative data, particularly regarding influenza strains and their relationship to vaccination history.

Several limitations should be considered when interpreting the results. Sample size: The study included only 26 participants, limiting statistical power and generalizability. Cross-sectional design: Samples were collected at a single time point, preventing assessment of temporal dynamics in antibody levels. Heterogeneity in vaccination and infection history: Variability among participants may have influenced antibody levels. The lack of functional assays, e.g., to analyse the neutralizing capacity of antibodies, was not directly assessed. These limitations highlight the need for larger, longitudinal studies incorporating functional immune assays.

Breast milk is a complex biological matrix containing lipids, proteins, and bioactive molecules that may interfere with immunoassays. The study addressed these challenges through method development, including centrifugation and optimisation of blocking conditions. Despite these efforts, matrix effects such as non-specific binding and lipid interference may still influence assay sensitivity and specificity. The use of endpoint titres rather than single-point absorbance measurements represents a strength, as it reduces the impact of such variability and provides a more robust quantitative assessment. The exclusion of IgM analysis in breast milk due to low detectable levels is consistent with known immunological principles, as IgM is primarily confined to the intravascular compartment and is not efficiently transported into secretions.

Future research should aim to conduct longitudinal studies to assess changes in antibody levels over time, include functional assays (e.g., virus neutralization) to determine protective capacity, investigate the impact of timing of maternal vaccination (pregnancy vs. lactation), expand antigen panels to include RSV and other emerging pathogens, and explore the relationship between breast milk antibodies and clinical outcomes in infants. Such studies will be essential for translating immunological findings into clinical recommendations.

In summary, this study supports the role of breast milk as a critical component of infant immune protection against respiratory viruses. The observed association between maternal systemic immunity and mucosal antibody transfer provides important insight into the mechanisms underlying passive immunity and highlights the potential of maternal vaccination as a strategy to protect vulnerable infants. The elevated levels of maternal serum IgG and breast milk IgA against respiratory viruses suggest a coordinated action between systemic and mucosal immunity, and that increasing maternal immunity through vaccination or prior infection may enhance infant protection. This pilot study provides a foundation for further research into maternal immunisation strategies and their role in preventing respiratory infections in early life.

## Data Availability

The data in study are available from the corresponding author upon reasonable request.
